# Global Trends in Proteome Remodeling of the Outer Membrane Modulate Antimicrobial Permeability in Klebsiella pneumoniae

**DOI:** 10.1128/mBio.00603-20

**Published:** 2020-04-14

**Authors:** Andrea Rocker, Jake A. Lacey, Matthew J. Belousoff, Jonathan J. Wilksch, Richard A. Strugnell, Mark R. Davies, Trevor Lithgow

**Affiliations:** a Infection and Immunity Program, Biomedicine Discovery Institute and Department of Microbiology, Monash University, Melbourne, Australia; b Doherty Department, at the Peter Doherty Institute for Infection and Immunity, The University of Melbourne and the Royal Melbourne Hospital, Melbourne, Australia; c Department of Microbiology and Immunology, at the Peter Doherty Institute for Infection and Immunity, The University of Melbourne and the Royal Melbourne Hospital, Melbourne, Australia; University of British Columbia

**Keywords:** antimicrobial resistance, porin, OmpK37, beta-barrel, carbapenem, carbapenems, porins

## Abstract

In Gram-negative bacteria, the permeability of the outer membrane governs rates of antibiotic uptake and thus the efficacy of antimicrobial treatment. Hydrophilic drugs like β-lactam antibiotics depend on diffusion through pore-forming outer membrane proteins to reach their intracellular targets. In this study, we investigated the distribution of porin genes in more than 2,700 *Klebsiella* isolates and found a widespread loss of OmpK35 functionality, particularly in those strains isolated from clinical environments. Using a defined set of outer-membrane-remodeled mutants, the major porin OmpK35 was shown to be largely responsible for β-lactam permeation. Sequence similarity network a*n*alysis characterized the porin protein subfamilies and led to discovery of a new porin family member, OmpK38. Structure-based comparisons of OmpK35, OmpK36, OmpK37, OmpK38, and PhoE showed near-identical pore frameworks but defining differences in the sequence characteristics of the extracellular loops. Antibiotic sensitivity profiles of isogenic Klebsiella pneumoniae strains, each expressing a different porin as its dominant pore, revealed striking differences in the antibiotic permeability characteristics of each channel in a physiological context. Since K. pneumoniae is a nosocomial pathogen with high rates of antimicrobial resistance and concurrent mortality, these experiments elucidate the role of porins in conferring specific drug-resistant phenotypes in a global context, informing future research to combat antimicrobial resistance in K. pneumoniae.

## Abstract (precis)

Klebsiella pneumoniae is a pathogen of humans with high rates of mortality and a recognized global rise in incidence of carbapenem-resistant K. pneumoniae (CRKP). The outer membrane of K. pneumoniae forms a permeability barrier that modulates the ability of antibiotics to reach their intracellular target. OmpK35, OmpK36, OmpK37, OmpK38, PhoE, and OmpK26 are porins in the outer membrane of K. pneumoniae, demonstrated here to have a causative relationship to drug resistance phenotypes in a physiological context. The data highlight that currently trialed combination treatments with a carbapenem and β-lactamase inhibitors could be effective on porin-deficient K. pneumoniae. Together with structural data, the results reveal the role of outer membrane proteome remodeling in antimicrobial resistance of K. pneumoniae and point to the role of extracellular loops, not channel parameters, in drug permeation. This significant finding warrants care in the development of phage therapies for K. pneumoniae infections, given the way porin expression will be modulated to confer phage-resistant—and collateral drug-resistant—phenotypes in K. pneumoniae.

## INTRODUCTION

Klebsiella pneumoniae is the causative agent of invasive and blood-borne infections and, as a prime example of carbapenem-resistant *Enterobacteriaceae* (CRE), it is regarded by the Centers for Disease Control and Prevention as an “urgent” threat to human health. These and related Gram-negative bacteria are prevalent in the environment and play an important role in soil ecosystems (1). However, in just a few decades K. pneumoniae has evolved from this innocuous existence to become a common and significant nosocomial pathogen (2). Initially associated only with the chronically unwell and immunocompromised individuals, *Klebsiella*’s proficiency at horizontal gene transfer has seen the rapid evolution of hypervirulent K. pneumoniae strains that infect even immunosufficient people (3, 4). High antibiotic selection pressure in hospitals and other environments precipitated the emergence of plasmid-mediated resistance, and *Klebsiella* now harbors antimicrobial resistance (AMR) phenotypes ranging from carbapenem resistance to colistin resistance, qualifying it as extremely drug resistant (5, 6). Until now, the most successful treatment regime for *Klebsiella* infections relied on antibiotics of the β-lactam type, particularly carbapenems. However, more and more *Klebsiella* strains are being identified with a growing diversity of β-lactamases, including the carbapenemases (7–9); carbapenem-resistant K. pneumoniae (CRKP) was first identified in China in 2007, and just 6 years later, carbapenem resistance was found in 13% of K. pneumoniae isolated from hospital patients across the country (10, 11).

Carbapenem resistance in *Klebsiella* has been observed in isolates confirmed to be carbapenemase negative (7, 12–17). A mutation in either of the genes *ompK35* and *ompK36* was also identified in these strains, leading to the suggestion that resistance is caused by the diminished import of carbapenem across the outer membrane, together with upregulation of β-lactamases such as AmpC cephalosporinases or extended-spectrum β-lactamases (ESBLs) (7, 12–17). The porins OmpK35 and OmpK36 belong to the Porin_1 (PF00267) group of bacterial outer membrane proteins. Both OmpK35 and OmpK36 form trimers composed of 16-stranded β-barrels integrated into the outer membrane, and the crystal structures of two of these proteins showed polar residues lining the internal pores (18). Structural and biophysical data agree that polar molecules of less than 600 Da in size would permeate the channels formed by OmpK35 and OmpK36 with limited selectivity (18–21). As most β-lactams are between 300 and 550 Da, they are believed to enter the periplasm via passive diffusion through these porins (22). In Escherichia coli, the homologous proteins OmpC and OmpF are designated “major porins” in the sense that they represent approximately half of the protein mass contributed by all β-barrel proteins in the outer membrane; they are so abundant across the outer membrane surface as to form large diffusion-limited arrays of pores that provide excellent permeability to small solutes (23–27).

As prominent surface molecules of K. pneumoniae, porins are involved in environmental sensing and nutrient acquisition. Correspondingly, loss of porin function may generate fitness costs due to impaired nutrient uptake (28–30) and to increased rates of phagocytosis and bacterial clearing (28, 31, 32) and correlates with decreased virulence as determined in mouse models of infection (28, 30, 31, 33). An increased abundance of OmpK26 or LamB has been observed in some clinical isolates of K. pneumoniae lacking the major porins (34–37), leading to suggestions that these pores might defray the fitness costs in terms of providing nutrient acquisition pores that do not also allow entry of antibiotics. Recently, similar suggestions were made regarding a third alternative porin in K. pneumoniae, OmpK37, which was suggested to possess a narrower channel to explain the observed lower diffusion rates of substrate molecules (38). Despite these interesting propositions, there remains limited knowledge of the antibiotic uptake through these various alternative porins, with their importance in antimicrobial resistance inferred indirectly from expression levels in clinical strains (34–37). Furthermore, there is a disagreement in the literature as to whether OmpK35 or OmpK36 is more important for clinical antibiotic resistance (17, 21, 28, 30, 39–41).

We sought to address three questions in this study. First, while loss-of-function mutations in *ompK35* and *ompK36* correlate with multidrug resistance, are they directly causative? Second, how prevalent are loss-of-function mutations in *ompK35* and *ompK36* around the world? Third, to what extent can remodeling of the outer membrane proteome impact antimicrobial resistance phenotypes? We analyzed all publicly available K. pneumoniae genome sequences and catalogued nonsense and missense mutations in the *ompK35* and *ompK36* genes, with population analysis suggesting that independent mutations have frequently occurred. Sequence similarity network analysis of this porin family revealed the existence of a new porin family member, OmpK38. To systematically address whether loss-of-function mutations in the major porin genes are causative for drug resistance, and to explore the idea that upregulation of other outer membrane pores may impact fitness or antibiotic sensitivity, we created an Δ*ompK35* Δ*ompK36* strain isogenic to a known clinical isolate. A series of strains were then generated in which either OmpK35, OmpK36, OmpK37, OmpK38, OmpK26, PhoE, or LamB was upregulated to be the major porin in the strain. Drug sensitivity, including sensitivity to carbapenems, was directly impacted by the identity of the porin expressed. This study provides evidence to highlight the consequences and causality of outer membrane proteome remodeling for AMR phenotypes in K. pneumoniae.

## RESULTS

### Global analysis of *Klebsiella* reveals major porin mutations.

**FIG 1:**
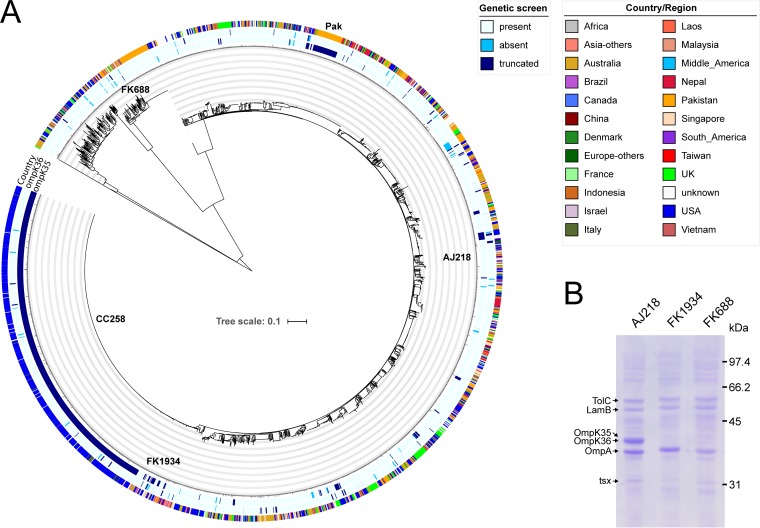
Global emergence of outer membrane remodeling in the Klebsiella pneumoniae species complex. (A) Maximum likelihood phylogenetic tree depicting the genome-wide sequence-based relationships of 2,706 publicly available *Klebsiella* genomes. The inner rings indicate intact gene sequences (light blue) and inactivating point mutations (dark blue) or deletions (cyan) in the *ompK35* and *ompK36* open reading frames. The outer rings show the country of isolation as detailed in the color key. “AJ218” designates the position of AJ218; “FK688” and “FK1934” designate the positions of FK688 and FK1934, respectively; “CC258” highlights the globally dominant CC258 *tonB79* subgroup, and the ST15 strains documented from a major hospital in Pakistan (42) are also designated. (B) Membrane preparations from AJ218, FK688, and FK1934 were subjected to analysis by SDS-PAGE and Coomassie blue staining of protein species. The identity of the major outer membrane proteins, determined by mass spectrometry, is indicated.

To assess the prevalence of loss-of-function mutations in the genes encoding OmpK35 or OmpK36, a global analysis was conducted on 2,706 publicly available genome data sets of *Klebsiella* (see Table S1 in the supplemental material). For OmpK35, premature stop codons were detected in 772 (29%) gene products (Table S1). These terminating mutations are found throughout the phylogeny but were enriched in the dominant lineage of K. pneumoniae CC258 and strains from a recent survey of a major hospital in Pakistan (42) (Fig. 1A). In contrast, mutations in *ompK36* were much less frequently observed (3.7%) and were randomly distributed across the global *Klebsiella* phylogeny (Fig. 1A). Similarly rare and random distributions of inactivating mutations were found in the other porin-encoding genes (Fig. S1).

10.1128/mBio.00603-20.2 FIG S1 Phylogenetic analysis of all *Klebsiella* general porins. Maximum likelihood phylogenetic tree depicting the genome-wide sequence-based relationships of 2,706 publicly available *Klebsiella* genomes. The rings show the presence of intact (light blue) and fragmented (dark blue) open reading frames of the indicated porins or their complete absence (blue). Download FIG S1, PDF file, 0.6 MB. Copyright © 2020 Rocker et al. 2020 Rocker et al. This content is distributed under the terms of the Creative Commons Attribution 4.0 International license.

10.1128/mBio.00603-20.5 TABLE S1 Genomics data summary. Download Table S1, XLSX file, 0.6 MB. Copyright © 2020 Rocker et al. 2020 Rocker et al. This content is distributed under the terms of the Creative Commons Attribution 4.0 International license.

Specific *tonB* alleles are used in multilocus sequence typing of K. pneumoniae, and the clonal *tonB79*-containing subgroup CC258 has a premature stop codon in *ompK35* (Fig. 1A). This group includes the K. pneumoniae ST258 and ST512 lineages characterized by the KPC carbapenemase. All of these strains possess the same mutation in *ompK35*, with a premature stop codon in place of amino acid position 88. Recent analysis suggested that ST258 has become one of the most successful multidrug-resistant bacterial pathogens in health care settings throughout the world (43). An insertion in loop 3 of OmpK36, previously proposed to contribute to the antimicrobial resistance of ST258 (30), is found in only 16% of these strains.

Within the global data set, mutations leading to a loss of OmpK35 were identified in 27.0% of isolates associated with infection, and in a further 13.6% of isolates from human carriage, pointing at an important association of *ompK35* inactivation with health care settings (Table S1). Furthermore, approximately 50% of OmpK36-deficient strains had an additional mutation in *ompK35* (49 of 99 strains), corroborating the global significance of genetic *ompK35* inactivation.

One such strain is *Klebsiella* FK688, recently isolated as the causative agent of a fatal, carbapenem-resistant sepsis (5). This isolate has an insertion in the 5′ end of *ompK35* that would change the promoter region and polypeptide sequence in the presumptive signal peptide, as well as a premature stop codon that was identified in the sequence data for *ompK36* (accession no. SRR11108934). The same study identified K. pneumoniae strain FK1934, which is phylogenetically distinct (Fig. 1A) yet also shows a lack of major porin expression (Fig. 1B) (accession no. SRR11108933). AJ218 is an isolate of K. pneumoniae from a human urinary tract infection (44, 45), with intact *ompK35* and *ompK36* genes and a substantial steady-state level of major porin in the outer membrane proteome (Fig. 1B and Table S2). In addition, AJ218 expresses the other known outer membrane proteins OmpA, TolC, LamB, and Tsx (Fig. 1B and Table S2). Comparing the outer membrane proteomes of these strains, remodeling is evident as steady-state levels of the major porins are undetectable in FK688 and FK1934 (Fig. 1B), correlating with their carbapenem resistance (5), with little obvious change in the abundance of any of the other proteins in the outer membrane proteome.

10.1128/mBio.00603-20.6 TABLE S2 Mass spectrometry data on most-abundant outer membrane proteins. Download Table S2, DOCX file, 0.01 MB. Copyright © 2020 Rocker et al. 2020 Rocker et al. This content is distributed under the terms of the Creative Commons Attribution 4.0 International license.

### Systematic assessment of AMR phenotype due to porin loss.

**FIG 2:**
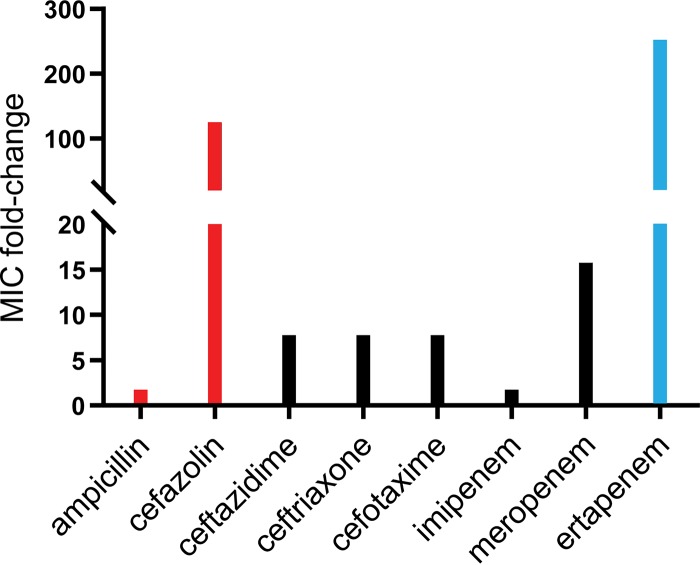
Effects of porin loss on antimicrobial resistance. Graphical representation of fold changes in the MICs between AJ218 and the porin-deficient isogenic derivative AJ218 Δ*ompK35* Δ*ompK36* depicted in Table 1. Red denotes a further increase in resistance that remains above the clinical breakpoint, while blue denotes an increase converting the strain from clinically sensitive to resistant.

To address the issue of causality between loss-of-function mutations in the major porins and carbapenem resistance, isogenic strains in the AJ218 background were constructed that were deleted for *ompK35*, *ompK36*, or both genes (the Δ*ompK35* Δ*ompK36* mutant). The single-knockout strains showed only relatively small changes in antibiotic susceptibility, with each of the porins reacting differently to different β-lactams (Table 1 and Fig. 2).

**TABLE 1:** MIC assessments of porin knockout strains

Drug	MIC (μg/ml)				
	AJ218	Δ*ompK35* mutant	Δ*ompK36* mutant	Δ*K35* Δ*K36* mutant	Breakpoint^*a*^
Ampicillin	4,096	4,096	8,192	8,192	≥32
Cefazolin	8	8	64	1,024	≥8
Ceftazidime	0.5	4	1	4	≥16
Ceftriaxone	0.125	0.125	0.5	1	≥4
Cefotaxime	0.0625	0.125	0.5	0.5	≥4
Imipenem	0.25	0.5	0.25	0.5	≥4
Meropenem	0.03125	0.03125	0.03125	0.5	≥4
Ertapenem	0.015625	0.03125	0.0625	4	≥2

a Clinical breakpoints as given in reference 97.

The porin-deficient Δ*ompK35* Δ*ompK36* strain showed increased resistance to all β-lactams, with a 256-fold increase in the MIC of the carbapenem ertapenem above the EUCAST and CLSI breakpoints for resistance in the Δ*ompK35* Δ*ompK36* strain (Table 1 and Fig. 2). The data also showed that porins provide a major entry route for the third-generation cephalosporins cefotaxime, ceftazidime, and ceftriaxone and the carbapenem meropenem (Table 1 and Fig. 2). Porin loss does not need to completely prevent carbapenem permeation but simply to reduce permeation rates to a point where very low rates of hydrolysis by other β-lactamases can control carbapenem levels in the periplasm. Typically, K. pneumoniae strains have a chromosomal gene encoding a β-lactamase called SHV-1 that is responsible for such intrinsic β-lactam resistance.

SHV-1-like β-lactamases (Bush-Jacoby group 2b) possess low-level activity against first-generation cephalosporins like cefazolin (46, 47). AJ218 encodes two variants of SHV-1, called SHV-44 and SHV-27: a single amino acid substitution in SHV-1 (R^205^L) gives rise to SHV-44 and does not greatly change the activity of the enzyme (48). Conversely, the single amino acid substitution (G^152^D) in the SHV-27 variant leads to increased activity in hydrolyzing cefotaxime (49). MICs of cefazolin increased from 8 μg/ml in the parent strain 128 times to 1,024 μg/ml in the Δ*ompK35* Δ*ompK36* strain (Table 1 and Fig. 2). Thus, loss of the major porins is both necessary and sufficient to make the Δ*ompK35* Δ*ompK36* strain highly resistant to this class of β-lactams. To further test the proposition that the SHV1-like β-lactamases are important in contributing to the observed carbapenem resistance, we made use of β-lactamase inhibitors tazobactam and avibactam (50, 51). Treatment with 4 μg/ml avibactam or 64 μg/ml tazobactam decreased the MIC for AJ218 of cefazolin 16- or 32-fold to 1 or 0.5 μg/ml (Table 2), respectively, indicating that SHV β-lactamases are contributing to the resistance in this strain. Conversely, addition of the β-lactamase inhibitors to ertapenem led to only a 2- to 4-fold decrease in the MIC for the Δ*ompK35* Δ*ompK36* strain. Taken together, the data indicate that β-lactamases play a minor role in making K. pneumoniae clinically resistant to ertapenem, whereas outer membrane remodeling exerts the major effect.

**TABLE 2:** MIC assessments with β-lactamase inhibitor combinations

Drug	MIC (μg/ml)					
	AJ218 plus empty vector			AJ218 Δ*K35* Δ*K36 *plus empty vector		
	No inhibitor	Tazobactam^*a*^	Avibactam^*b*^	No inhibitor	Tazobactam^*a*^	Avibactam^*b*^
Ampicillin	8,192	4	4	8,192	1,024	32
Cefazolin	8	0.5	1	1,024	256	16
Ceftazidime	0.5	0.125	0.25	4	1	1
Ceftriaxone	0.0625	0.03125	0.0625	0.5	0.5	0.5
Cefotaxime	0.0625	0.03125	0.0625	0.5	0.5	0.5
Imipenem	0.5	0.25	0.25	0.5	0.25	0.5
Meropenem	0.03125	0.015625	0.03125	0.5	0.5	0.0625
Ertapenem	0.015625	0.008	0.015625	4	2	1

a Tazobactam was used at a final concentration of 64 μg/ml.

b Avibactam was used at a final concentration of 4 μg/ml.

### OmpK38 is a new member of the general bacterial porin family.

**FIG 3:**
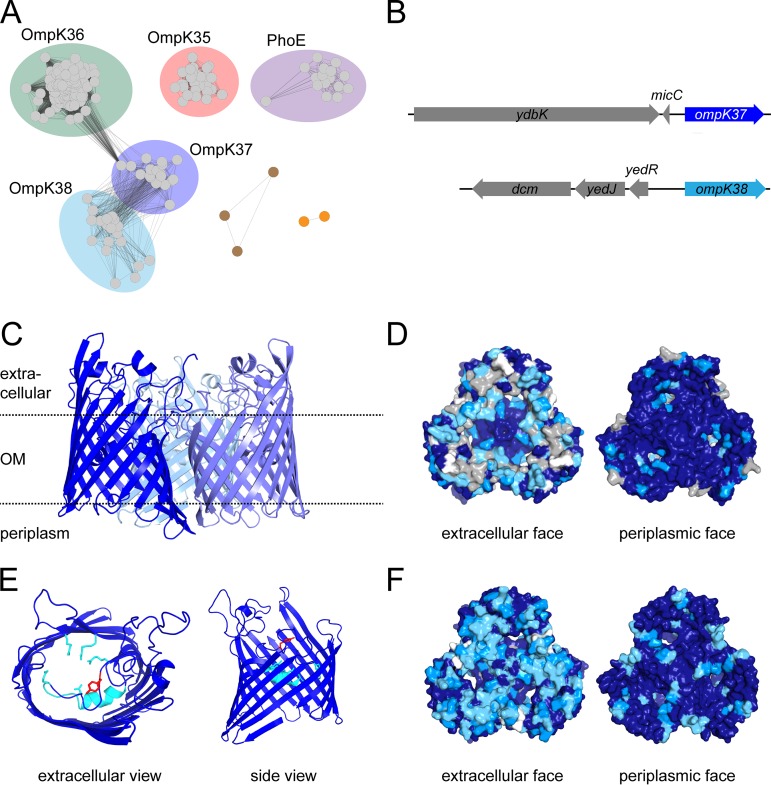
OmpK37 is a member of the general bacterial porin family. (A) Sequence similarity networks graphically depict the homology between all Porin_1 (PF00267) sequences within the genus *Klebsiella* (NCBI taxID 570) available in UniProt (release 2019_02). Each dot represents the sequence of a protein species. Dark lines between sequences represent similar sequences, and lighter lines represent less similar sequences with a minimal alignment score of 138. The analysis shows distinct subclasses for OmpK35 (red) and PhoE (purple) homologues, as well as the close relationships between OmpK36 (green), OmpK37 (dark blue), and OmpK38 (light blue) subclasses. Brown dots depict individual homologous gene transfers of porins from *Proteus*/*Morganella* species into *Klebsiella* strains, while the orange dots represent a putative porin subfamily found in a subset of Klebsiella variicola genomes. (B) Gene synteny definitions for the genes encoding OmpK37 and OmpK38. (C) The structure of OmpK37 determined by X-ray crystallography (PDB ID 6V78), showing the typical trimeric arrangement of monomers. Each monomer adopts a β-barrel fold and is colored a shade of blue. The position of the protein within the outer membrane (OM) and the extracellular and periplasmic sides are indicated. (D) Sequence similarity of OmpK37 and OmpK36 from AJ218, ranging from identical (dark blue) to medium (blue and light blue) and low (gray), mapped onto the surface representation of OmpK37 viewed from the extracellular (left) or periplasmic (right) side of the outer membrane. White patches represent deletions/insertions in the loop regions. (E) Closeup view of the constriction zone (light blue), forming the narrowest point within the pore and contributing to substrate selection by size and charge filtering. The infolded loop 3 is highlighted in light blue. The side chain of Tyr_118_ (red) is pointing toward the extracellular pore entrance. (F) Sequence similarity of OmpK37 and OmpK38 from AJ218, colored as in panel C.

In the protein classification system organized by Pfam, OmpK35 and OmpK36 belong to the Porin_1 (PF00267) group of bacterial outer membrane proteins together with the anion-selective porin PhoE, which adopt a conserved protein structure as shown by X-ray crystallography (18, 20, 52). A fourth porin in *Klebsiella*, OmpK37, is also a member of this family. Sequence-based clustering of PF00267 for all proteins from the genus *Klebsiella* revealed that OmpK35 and PhoE porins cluster into clearly distinct subfamilies (Fig. 3A). In contrast, OmpK36 porins show sequence similarities to the OmpK37 subfamily, which therefore cluster together. Surprisingly, this analysis demonstrated a further separation of what were annotated as OmpK37 homologs into two separate subgroups (Fig. 3A, depicted in light and dark blue). Sequence analysis of the model K. pneumoniae AJ218 genome revealed the presence of two *ompK37*-like genes, each with a defined gene synteny conserved in other *Klebsiella* genome contexts (Fig. 3B). Both *ompK37*-like genes were identified in nearly all 2,706 strains in the phylogeny (97.8% and 98.3%, respectively) (Table S^1^ and Fig. S^1^). We designate the novel porin family OmpK38, suggesting that gene synteny should be used as the basis for distinguishing these genes in genome sequencing projects.

### Structure-based comparisons of *Klebsiella* porins.

We solved the structure of OmpK37 by X-ray crystallography, providing a structural map at 2.6-Å resolution to better understand the relationship of OmpK37 and OmpK38 to the major porins (Table 3 and Fig. 3C). Like the other porins, OmpK37 assembles into a trimer, with each monomeric unit composed of 16 β-strands that cross the outer membrane to form a β-barrel (Fig. 3C). All sequence variation seen within and between OmpK36 and OmpK37 trimers maps to the extracellular loops of the β-barrels and residues lining the upper reaches of the pore (Fig. 3D). The influence of these loops on the permeation of small molecules is unknown. Analysis of the structure also illustrates an important feature of the β-barrel porins in terms of the solute channels that they form: no clear path can be seen through the pore, due to the channel being particularly constrained in the central region of the protein (Fig. 3D). This impediment is dominated by the inward folding of extracellular loop 3 into the β-barrel lumen, to form a “constriction zone” (Fig. 3E). This feature has been shown to impact the relative diffusion rates of solutes through a porin (20, 53, 54). In this loop 3, OmpK37 carries a bulky residue (Tyr^118^) not found in OmpK36, a residue that was previously speculated to lead to a decreased pore diameter (38). However, the crystal structure of OmpK37 dismisses this conjecture as the tyrosine side chain is oriented toward the pore exit such that it does not contribute to the constriction zone (Fig. 3E). All other residues of the constriction zone in OmpK37 (Arg^37^, Arg^75^, Arg^126^, Lys^16^, Asp^106^, and Glu^110^) are conserved and adopt the same conformation as in OmpK35 and OmpK36.

**TABLE 3:** Data collection and refinement statistics

Statistic	Value for OmpK37 (6V78)
Data collection	
Space group	P2_1_2_1_2
Cell dimension	
*a, b, c* (Å)	109.52, 138.51, 91.70
α, β, γ (°)	90, 90, 90
Resolution (Å)	50–2.6 (2.7–2.6)
*R*_meas_	19.3 (176.4)
I/σI	11.11 (1.73)
Completeness (%)	99.2 (98.8)
Redundancy	7.4 (7.5)
CC_1/2_	99.7 (69.2)

Refinement	
Resolution (Å)	49.3–2.6
No. of reflections	43,308
*R*_work_/*R*_free_	25.7/32.3
No. of atoms	
Protein	8,382 (trimer)
Water	73
*B*-factor	
Protein	56.3
Water	47.2
RMS^*a*^ deviations	
Bond lengths (Å)	0.008
Bond angles (°)	1.08
Ramachandran statistics (%)	
Preferred	95.9
Allowed	4.1
Outliers	0

a RMS, root mean square.

To complete the comparative analysis of pore properties, we calculated an *in silico* model of OmpK38 based on the published high-resolution structure of OmpK36 (PDB ID 5O79). The modeled OmpK38 closely resembles the structure of OmpK36 in the β-barrel domain, with variations observed only in the external loops. This is in agreement with the observation that the sequence similarity is high for residues located at the periplasmic surface, while residues in the extracellular loops are more variable (Fig. 3F).

**FIG 4:**
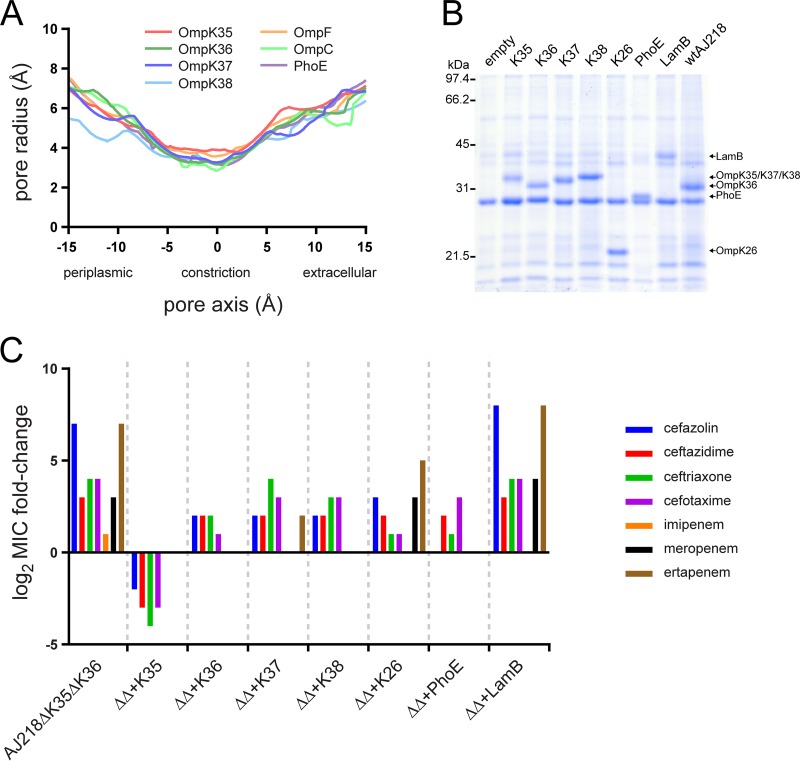
Effects of alternative porin expression in the *Klebsiella* outer membrane. (A) Pore radius analysis of various structurally determined porins (OmpK35 [5O77], red; OmpK36 [5O79], dark green; OmpK37 [6V78], dark blue; OmpF [2OMF], orange; OmpC [2J1N], light green; PhoE [1PHO], purple) and the *in silico* model of OmpK38 (light blue) as calculated by HOLE (94). All porins display a similar pore trajectory with only minor variations of the pore radius along the entire axis of the pore. At the constriction zone, the narrowest point along the pore, OmpF and OmpK35 show a slightly larger pore radius than OmpC/OmpK36, while OmpK37, OmpK38, and PhoE are comparable to the latter. (B) Membrane preparations from wild-type AJ218 (wt AJ218) and AJ218 Δ*ompK35* Δ*ompK36* (ΔΔ) strains expressing the indicated porins from an anhydrotetracycline-inducible promoter. The extracts were subjected to SDS-PAGE and Coomassie blue staining. (C) Graphical representation of the drug resistance data in Table 5. Log_2_ fold change of the MIC values of the indicated porin-expressing strains are given compared to wild-type MIC levels.

For β-lactams, the pore characteristics of the major entry route across the outer membrane will determine how readily an effective concentration of drug can equilibrate in the periplasm, in order to inhibit its target. The structural frameworks of various porins are largely superimposable: the diameter of the entry and exit pore sizes are essentially the same, with crystal structures showing key differences in the length of loops 3, 4, 5, and 6, with the loop 3 differences changing aspects of the constriction zone geometry (18, 20, 52). Comparing the pore trajectories and sizes of the determined crystal structures (Fig. 4A) revealed that OmpK35 porins show a slightly larger constriction zone radius, while OmpK36 porins are slightly more constricted, as previously proposed (20). PhoE porins have a constriction zone radius comparable to OmpK36, while OmpK37 is in between the OmpK35 and OmpK36 pores (Table 4). The pore trajectory and constriction zone radius of the OmpK38 model more closely match OmpK36 than OmpK37 (Fig. 4A and Table 4).

**TABLE 4:** Constriction zone radii (Å) of porin structures

Porin (PDB_ID)	Species	Radius (Å) determined by:		
		BetaCavity	MOLEonline	HOLE
OmpK37 (6V78)	*K. pneumoniae*	3.42	3.39	3.26
OmpC (2J1N)	*E. coli*	3.05	3.05	2.84
OmpK36 (5O79)	*K. pneumoniae*	3.34	3.22	3.17
OmpF (2OMF)	*E. coli*	3.73	3.67	3.56
OmpK35 (5O77)	*K. pneumoniae*	3.97	3.77	3.78
OmpK38 (*in silico*)	*K. pneumoniae*		3.20	3.16
PhoE (1PHO)	*E. coli*	3.30	3.26	3.13

### The effect of outer membrane proteome remodeling on β-lactam permeation.

To determine whether these structural differences are meaningful for antimicrobial sensitivity, the porin-deficient Δ*ompK35* Δ*ompK36* strain was engineered to express each of the other porins under the control of a heterologous promoter, to increase their expression level to become “major” porins (Fig. 4B). In rich growth media, the cross-complemented strains grow at rates equivalent to the isogenic AJ218 strain (Fig. S2), with doubling times and final cell densities of the individual strains comparable over 24 h under conditions equivalent to those used for MIC experiments.

10.1128/mBio.00603-20.3 FIG S2 Growth analysis of porin-expressing strains. AJ218 Δ*ompK35* Δ*ompK36* expressing the indicated porins from an anhydrotetracycline-inducible promoter was cultured for up to 24 h, and cell density was monitored by measuring absorption (OD_600_) over time. Error bars depict the standard deviation for biological triplicates. Growth rates and final cell densities of all strains were comparable. Download FIG S2, PDF file, 0.2 MB. Copyright © 2020 Rocker et al. 2020 Rocker et al. This content is distributed under the terms of the Creative Commons Attribution 4.0 International license.

High-level expression of OmpK35 was found to sensitize *Klebsiella* to both penicillins and cephalosporins, indicating that OmpK35 provides the major influx pathway for these compounds (Table 5 and Fig. 4C). The MIC values are even lower in this strain than in the wild type, with the OmpK35 expression levels 7.7-fold higher upon induction with anhydrotetracycline (ATc) than in the wild type (Fig. S3). In contrast with the expression of OmpK35, MIC values of the OmpK36-expressing strain are restored for the carbapenems (meropenem and ertapenem) but not for the cephalosporins (Table 5). This indicates that while carbapenems diffuse through either OmpK35 or OmpK36 equally well, cephalosporins favor the channel in OmpK35.

**TABLE 5:** MIC assessments of porin-expressing strains

Drug	MIC (μg/ml) for:								
	AJ218, empty vector	AJ218 Δ*K35* Δ*K36* plus:							
		Empty vector	OmpK35	OmpK36	OmpK37	OmpK38	OmpK26	PhoE	LamB
Ampicillin	>2,048	>2,048	512	>2,048	>2,048	>2,048	>2,048	>2,048	>2,048
Carbenicillin	>2,048	>2,048	1,024	>2,048	>2,048	>2,048	1,024	>2,048	>2,048
Cefazolin	4	512	2	16	16	16	32	4	1,024
Ceftazidime	0.25	2	0.03125	1	1	1	1	1	2
Ceftriaxone	0.03125	0.5	0.002	0.125	0.5	0.25	0.0625	0.0625	0.5
Cefotaxime	0.03125	0.5	0.004	0.0625	0.25	0.25	0.0625	0.25	0.5
Imipenem	0.25	0.5	0.25	0.25	0.25	0.25	0.25	0.25	0.25
Meropenem	0.03125	0.25	0.03125	0.03125	0.03125	0.03125	0.25	0.03125	0.5
Ertapenem	0.015625	2	0.015625	0.015625	0.0625	0.015625	0.5	0.015625	4

10.1128/mBio.00603-20.4 FIG S3 Western blot analysis of OmpK35 expression. Western blotting detecting OmpK35 levels in AJ218 or AJ218 Δ*ompK35* Δ*ompK36* (ΔΔ) carrying pJP-Cm, pJP(OmpK35), or pJP(OmpK36). The antibody was raised against E. coli OmpF but shows cross-reactivity to *Klebsiella* OmpK35 and, to a lesser degree, OmpK36. Densitometry showed a 7.7-fold increase in OmpK35 expression levels in AJ218 Δ*ompK35* Δ*ompK36* carrying pJP(OmpK35) compared to the wild-type strain. Download FIG S3, DOCX file, 2.2 MB. Copyright © 2020 Rocker et al. 2020 Rocker et al. This content is distributed under the terms of the Creative Commons Attribution 4.0 International license.

Comparing the effects of OmpK37 and OmpK38 to the related porin OmpK36 was revealing. OmpK37 has a selectively lower permeability to ertapenem, ceftriaxone, and cefotaxime than OmpK36. While OmpK38 matches the permeation properties of OmpK37 nearly exactly, it is characterized by a higher permeability to ertapenem, reaching MIC values similar to strains expressing OmpK36. The structural similarities suggest that it is not the pore size, but perhaps the physicochemical properties of the pore surface (20), that would impact these selective differences in drug sensitivity.

PhoE, OmpK26, and LamB are considered substrate-specific porins. This expectation was evidenced in the strain expressing LamB as its dominant pore: there was no change in the MIC values, indicating that LamB acts as a substrate (maltose and maltodextrin)-specific porin that does not allow significant permeation by β-lactam antibiotics (Table 5 and Fig. 4C). The converse was true for PhoE, which enables the influx of most β-lactams as readily as OmpK36, and for OmpK26, which provides permeability to all tested cephalosporins (Table 5 and Fig. 4C).

### Do the various pore types play a role in restricting the permeability for other drugs?

Sensitivity to the β-lactamase inhibitors avibactam and tazobactam was also assessed. Both inhibitors possess an intrinsic low-level antimicrobial activity (55, 56), which leads to bacterial killing in MIC experiments. Differences in inhibitor uptake were observed (Table 6), including that upregulation of OmpK26 or OmpK35 sensitized the Δ*ompK35* Δ*ompK36* strain to tazobactam but not to avibactam, while OmpK36, OmpK37, and PhoE seem to provide preferential entry routes for avibactam.

**TABLE 6:** MIC assessments for β-lactamase inhibitors

Drug	MIC (μg/ml) for:								
	AJ218, empty vector	AJ218 Δ*K35* Δ*K36* plus:							
		Empty vector	OmpK35	OmpK36	OmpK37	OmpK38	OmpK26	PhoE	LamB
Tazobactam	256	512	128	256	512	512	64	512	512
Avibactam	>256	>256	256	128	128	64	>256	128	256

Imipenem is a relatively hydrophobic zwitterionic carbapenem with a neutral net charge and the smallest β-lactam tested (molecular weight, 299 Da). It has been suggested that the combination of imipenem-relebactam with an aminoglycoside may be a promising approach for isolates with reduced susceptibility to imipenem-relebactam (57). To address the drug resistance to non-β-lactam drugs, the Δ*ompK35* Δ*ompK36* strain and the porin-complemented strains were subjected to further MIC testing.

Only 2-fold differences in MIC values between the wild-type and porin-deficient strains were observed for gentamicin, neomycin, tobramycin, and spectinomycin (Table S3), indicating that diffusion of aminoglycosides through porins is of minor importance in *Klebsiella*. Quinolones are thought to be able to diffuse both through the outer membrane layer and through the porin channels (58, 59). However, only 2-fold differences in MIC values between the wild-type and porin-deficient strain are observed for the fluoroquinolone ciprofloxacin and for the more hydrophobic nalidixic acid. Like these other hydrophobic compounds, the large (734-Da) macrolide erythromycin has been suggested to permeate the outer membrane via the lipid bilayer (60, 61). This theory is supported by the limited (i.e., 2-fold) change in antibiotic sensitivity (Table S3). A sensitization to antibiotics is seen in the strain expressing OmpK35 as the major porin, indicating that OmpK35 is permeable to aminoglycosides, quinolones, and macrolides. Relative MIC values revealed that the expression of other porins has only minor effects, while LamB has no effect on modulating the drug sensitivity of the porin-deficient strain. The antimicrobial peptide polymyxin B binds to the lipopolysaccharide (LPS) layer to exert its antimicrobial activity (62). As expected, no difference in MIC was observed for the tested strains. The data confirm that mutations inducing porin loss will not greatly contribute to resistance phenotypes against antibiotics other than β-lactams.

10.1128/mBio.00603-20.7 TABLE S3 MIC assessments for other drug classes. Download Table S3, DOCX file, 0.02 MB. Copyright © 2020 Rocker et al. 2020 Rocker et al. This content is distributed under the terms of the Creative Commons Attribution 4.0 International license.

## DISCUSSION

### The prevalence of outer membrane proteome remodeling in response to drug treatment.

A major finding of this study comes from comparing carbapenem susceptibility profiles for AJ218 and the isogenic Δ*ompK35* Δ*ompK36* strain. This indicated that porin loss alone can confer carbapenem resistance in *Klebsiella.* Given the preponderance of *ompK35* mutations globally (Fig. 1A), the apparent ease with which additional *ompK36* mutations can be tolerated in these strains, and the presence of chromosomal SHV β-lactamases in the core genome of K. pneumoniae, this carbapenemase-negative and ESBL-negative CRKP phenotype too will be global.

The experiments provide a further significant finding. In combination treatment, tazobactam addition is less effective against the Δ*ompK35* Δ*ompK36* strain, while avibactam shows superior effectiveness. Tazobactam is itself a β-lactam compound of 300 Da in size (63), and it appears to be dependent on porins for uptake into the periplasm. While of similar overall size (265 Da), avibactam is not a β-lactam but a bridged diazabicyclo[3.2.1]octanone (51, 64), and our data showed that its permeation into the periplasm of the Δ*ompK35* Δ*ompK36* strain is more effective than for tazobactam, i.e., that it likely can cross the outer membrane via channels other than OmpK35 and OmpK36 (Table 6). Recent successful combination treatments with meropenem and the β-lactamase inhibitor vaborbactam (65) might also be effective on porin-deficient K. pneumoniae, given that vaborbactam is a compound based on a cyclic boronic acid pharmacophore (66, 67) and, at 267 Da in size, it may access the periplasm via porins other than OmpK35 and OmpK36.

Our current knowledge on regulation of porin gene expression in K. pneumoniae is minimal. Studies support the hypothesis that OmpK35 is thus the main contributor to antibiotic uptake in *Klebsiella* (40, 41), consistent with the observation that mutations in *ompK35* are more prevalent globally in parallel with the rise in β-lactamase resistance (Fig. 1A), and that OmpK35 is dispensable for growth and virulence in the human niche (30). In E. coli, expression of the OmpK35 and OmpK36 homologs, OmpF and OmpC, respectively, is regulated so as to have one or the other (but not both) expressed at high levels (68). This switching between expression of the major porins is regulated by small RNAs which can be dysregulated in the presence of β-lactam drugs (69). While little is known about the regulation of expression of *ompK35*, *ompK36*, *ompK37*, and *ompK38* in K. pneumoniae, a study in Klebsiella aerogenes shows that overexpression of the small RNAs MicF and MicC can suppress expression of *omp35* and *omp36*, respectively (70). Another study reported that *ompK37* expression is induced in response to a loss of the major porins (38). Our results with upregulation of different major porins (Table 5 and Fig. 4C) give credence to the hypothesis that changes in gene expression levels induced during growth in human tissues including serum would modulate drug sensitivity. We suggest further studies are warranted to measure the expression levels of porins in clinical isolates grown in human serum, to determine which of the *ompK35*, *ompK36*, *ompK37*, or *ompK38* genes would be most active under those conditions.

### The different β-barrel pores and features of outer membrane proteome remodeling.

LamB is known as a transporter of maltodextrins and efficiently transports sugar polymers. Overexpression of this porin in knockout strains does not change the obtained MIC values, indicating that the substrate specificity of LamB is too narrow for it to efficiently transport β-lactam antibiotics. Conversely, the β-lactam MICs of PhoE-expressing strains are very similar to the parental AJ218 with the exception of ceftazidime and cefotaxime. This indicates that the PhoE channel is largely equivalent to the major porins for the influx of most β-lactam antibiotics. OmpK26 has been reported to be overexpressed in porin-deficient hospital isolates selected by carbapenem treatment (34, 36, 37). In our study, the strain expressing OmpK26 as its major porin is relatively impermeable to carbapenem, which is consistent with the observation that prolonged carbapenem treatment might select for increased *ompK26* expression (34, 36, 37). Importantly, AJ218 expressing OmpK26 as its major porin showed MIC values similar to a porin-expressing wild-type strain for the tested third-generation cephalosporins. While there is no clear structure-based rationale for these observations, the MIC data suggest that OmpK26-expressing strains should be treated with cephalosporins.

OmpK37 and OmpK38 are homologous to OmpK36, showing ∼70% identity at the amino acid sequence level. In a previous report, OmpK37 was speculated to have a narrower pore, based on a lower rate of sugar influx and increased antimicrobial resistance (38). Our results show a lower permeability to some antibiotics (ertapenem, cefotaxime, and ceftazidime), but the structure of OmpK37 reported here demonstrates that the pore diameters do not differ and could not determine drug specificity. To model porin permeability, Acosta-Gutiérrez et al. (20) highlighted the importance of the size and charge of the constriction zone for drug permeation between different Gram-negative major porins. While these properties may be important for the differences between some of the *Klebsiella* porins observed in this study, the residues within the constriction zone of OmpK36, OmpK37, and OmpK38 are largely identical. Since differences in drug uptake were observed between strains expressing these related porins, we suggest that variation in the external loops acts as a crucial determinant of antibiotic permeability. It remains unclear what the selection pressure is for this variation. One possibility is host factors involved in driving antigenic variation for protection against antibodies and/or complement, as OmpK36 has been shown to be targeted by components of the complement pathway (71–73). Another possibility, and not mutually exclusive, is that selection for variation in OmpK36, OmpK37, and OmpK38 is being driven by microbial factors, such as bacteriophages or colicins (74, 75). Bacteriophages such as GH-K3 that use OmpK36 as a receptor would produce the selective pressure for the evolution of phage-resistant *ompK36* mutants (74), which would select for a collateral drug resistance phenotype given the MIC data in our study. Given prospects for phage therapy to treat CRE infections, the evolutionary drivers on outer membrane protein remodeling in K. pneumoniae and other CRE pathogens need further attention.

## MATERIALS AND METHODS

### Chemicals and reagents.

Ampicillin, carbenicillin, and tetracycline were purchased from Astral Scientific. Avibactam was purchased from Selleck Chemicals. All other antibiotics, including tazobactam, were purchased from Sigma-Aldrich in the highest possible grade.

### Comparative genomics.

To examine the distribution of porin proteins in *Klebsiella*, a database of 2,706 publicly available genome sequences was constructed (42, 76) (see Table S1 in the supplemental material). All genomes that required assembly were assembled using Skesa v2.3.0 (77), and chromosomal sequence types were determined for each genome assembly using the genotyping toolkit Kleborate (https://github.com/katholt/Kleborate), which is aligned to the BIGSdb-Kp multilocus sequence typing (MLST) scheme (78). For visual purposes, a single nucleotide polymorphism (SNP) midpoint-rooted maximum-likelihood phylogenetic tree was generated. Genomes were mapped to the K. pneumoniae reference strain NTUH-K2044 (GenBank accession no. AP006725.1 [79]) using minimap2, and SNPs were called using snippy v4.3.9 (https://github.com/tseemann/snippy). Phylogenetic inference was performed using IQ-TREE v1.6.10 (80) using the GTR+F+G4 model based on 4,285 parsimonious SNPs and 1,000 ultrafast bootstraps (81). Porin variants were identified in the 2,706 draft genome assemblies by a BLASTN screening tool (82) applying the cutoffs 80% identity and 90% reference length. Hits were translated into protein sequence for identification of putative premature stop codons. Images were made using Interactive Tree Of Life (iTOL) v4 (83).

Whole-genome sequences were generated for FK688 and FK1934 using Illumina short-read genome sequencing on the Illumina NextSeq 500 platform with 150-bp paired-end reads. Libraries were generated using the Illumina Nextera XT DNA sample preparation kit.

Protein sequences belonging to Pfam family Porin_1 (PF00267) and the genus *Klebsiella* (NCBI taxID 570) were downloaded from UniProt (release 2019_02). Sequence similarities were calculated using EFI-EST (84), performing an all-by-all BLAST search and clustering based on a minimal pairwise alignment score of 138. Sequence similarity networks were visualized using Cytoscape 3.7.1 (85).

### Bacterial strains and cultures.

An overview of the strains and plasmids used in this study is given in Table S4. Knockout strains were constructed using the “gene gorging” technique (45, 86), described in detail in Text S1. The cloning of plasmids used for protein expression in E. coli or K. pneumoniae is described in Text S1.

10.1128/mBio.00603-20.1 TEXT S1 Supplemental methods. Download Text S1, DOCX file, 0.03 MB. Copyright © 2020 Rocker et al. 2020 Rocker et al. This content is distributed under the terms of the Creative Commons Attribution 4.0 International license.

10.1128/mBio.00603-20.8 TABLE S4 Strains, plasmids, and oligonucleotides. Download Table S4, DOCX file, 0.02 MB. Copyright © 2020 Rocker et al. 2020 Rocker et al. This content is distributed under the terms of the Creative Commons Attribution 4.0 International license.

### Structure determination and modeling.

OmpK37 was overexpressed and purified from E. coli C41 cells as described in Text S1. Initial crystallization conditions were screened at the Monash Molecular Crystallization Facility using commercially available screens. Following optimization, crystals of OmpK37 were grown using a sitting drop vapor diffusion setup with a reservoir solution of 125 mM SPG buffer (2:7:7 succinic acid-sodium hydrogen phosphate-glycine) (pH 4.5) and 20% (wt/vol) polyethylene glycol 1,500 and directly flash-frozen in liquid nitrogen. Diffraction data were collected at 100 K at the Australian synchrotron and processed with the XDS software package (87) in the space group P2_1_2_1_2 to 2.6-Å resolution. Five percent of the reflections were randomly selected for calculation of *R*_free_ and inherited to all data sets. Initial phases were obtained by molecular replacement using Phaser for MR (88) and the published structure of the OmpK36 porin (1OSM) trimmed of its external loops as a search model. The initial model was improved in iterative cycles of manual building in Coot and refinement using Phenix (89, 90). Final structure validation was performed using MolProbity (91).

Structure prediction of an OmpK38 model was performed using the mini.rosetta threading protocol as implemented in the Rosetta software package, using OmpK36 (5O79) as a reference structure. This was followed by an energy minimization using the relax protocol in Rosetta.

Pore geometries of OmpK37 (6V78) and published structures of OmpC (2J1N), OmpK36 (5O79), OmpF (2OMF), OmpK35 (5O77), and PhoE (1PHO) were analyzed using BetaCavity (92), MOLEonline (93), and HOLE (94). Figures were prepared using PyMOL (95).

### Outer membrane protein analysis.

Overnight cultures of strains harboring porin expression plasmids were diluted 1:50 in cation-adjusted Mueller-Hinton Broth containing chloramphenicol (CaMHB-Cm) and grown at 37°C, 200 rpm, until an optical density at 600 nm (OD_600_) of 0.5 was reached. Subsequently, cultures were diluted to an OD_600_ of 0.0005 in CaMHB-Cm, induced with the appropriate amount of ATc (empty = 400 ng/ml, K35 = 150 ng/ml, K36 = 200 ng/ml, K37 = 10 ng/ml, K26 = 100 ng/ml, PhoE = 5 ng/ml, LamB = 400 ng/ml), and grown at 37°C, 200 rpm, for 4.5 or 20 h. At these time points, 14 ml (4.5 h) or 2 ml (20 h) of culture was harvested by centrifugation (4,500 × *g*, 4°C, 10 min) and resuspended in 1.8 ml of buffer A (50 mM Tris, 150 mM NaCl, 5 mM EDTA, pH 7.5). Cells were broken by sonication on ice (5 × 10 s, amplitude 2, duty 100%) and centrifuged at 2,000 × *g* to remove cell debris. Sodium lauroyl sarcosinate was added to the supernatant to a final concentration of 0.5% (wt/vol), and the mixture was incubated for 30 min on ice before centrifugation at 25,000 × *g* at 4°C for 30 min. The pellet containing the outer membranes was resuspended in 200 μl of buffer B (25% [wt/vol] sucrose, 50 mM Tris, 5 mM EDTA, pH 7.5) and loaded onto an SDS-polyacrylamide gel containing 11% (wt/vol) 37.5:1 acrylamide-bisacrylamide, 0.375 M Tris (pH 8.8), 0.2% (wt/vol) sodium dodecyl sulfate, and 0.5 mM EDTA in the separating gel and 4% (wt/vol) 37.5:1 acrylamide-bisacrylamide, 0.25 M Tris (pH 6.8), 0.1% (wt/vol) sodium dodecyl sulfate, and 0.5 mM EDTA in the stacking gel.

### MIC determination.

MICs were determined and interpreted using the broth microdilution method outlined by the Clinical and Laboratory Standards Institute (96).

### Data availability.

The X-ray structure of OmpK37 has been deposited in the PDB with the accession code 6V78. Genome sequence data for Klebsiella quasipneumoniae subsp. *similipneumoniae* (FK688) and Klebsiella pneumoniae (FK1934) have been deposited at the NCBI under the BioProject ID PRJNA607402 with Sequence Read Archive codes SRR11108934 (FK688) and SRR11108933 (FK1934).
