# Supplementary material for: Global Trends in Proteome Remodeling of the Outer Membrane Modulate Antimicrobial Permeability in Klebsiella pneumoniae
Source: mBio. 2020 Apr 14;11(2):e00603-20. doi: 10.1128/mBio.00603-20 (PMC7157821; doi:10.1128/mBio.00603-20)

Supplementary Figure S1 – Phylogenetic analysis of all *Klebsiella* general porins. Maximum likelihood phylogenetic tree depicting the genome-wide sequence-based relationships of 2,706 publicly available *Klebsiella* genomes. The rings show the presence of intact (light blue) and fragmented (dark blue) open reading frames of the indicated porins or their complete absence (blue).

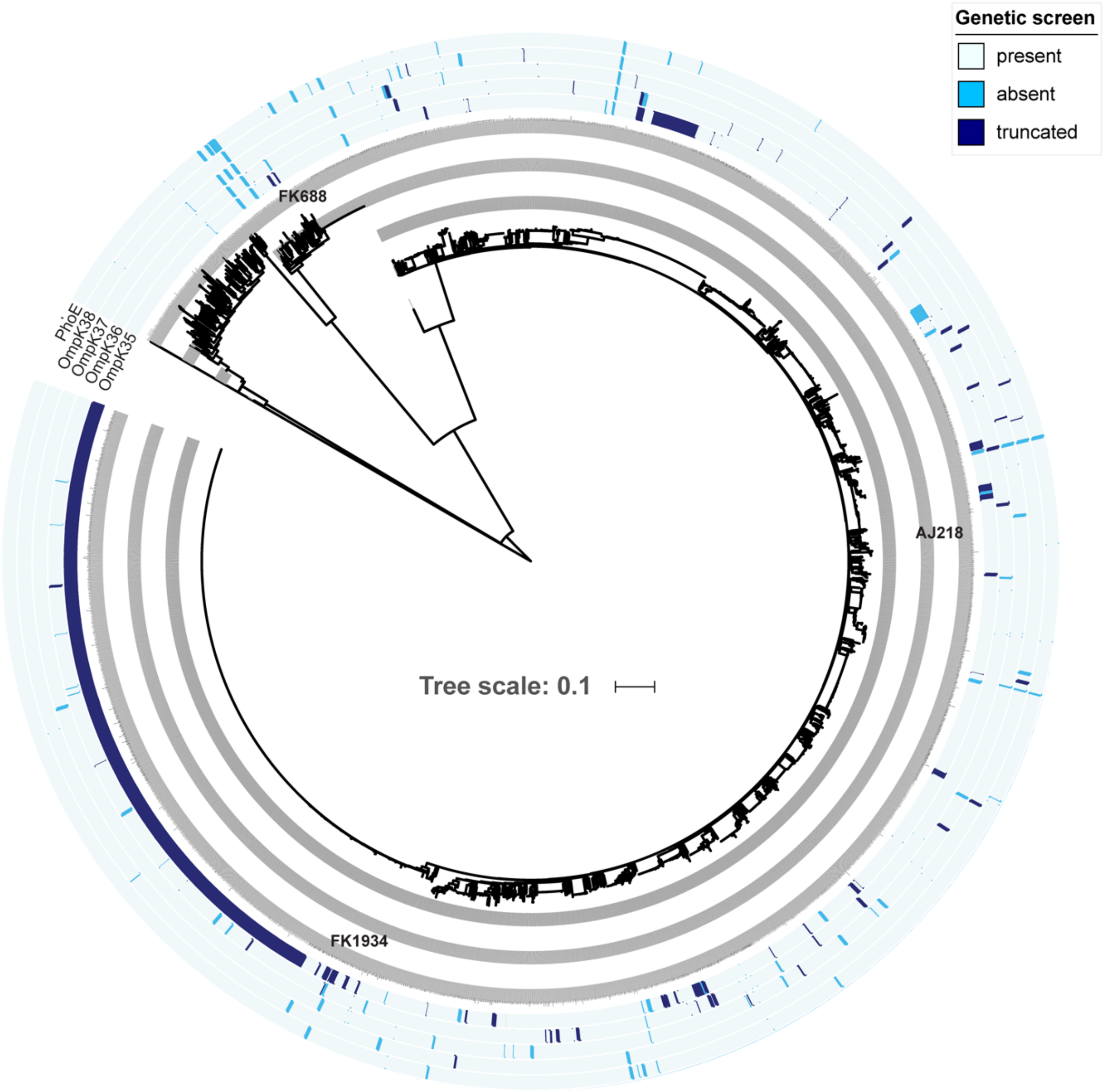

Supplement: FIG S1 [file mBio.00603-20-sf001.pdf]
