# Supplementary material for: Global Trends in Proteome Remodeling of the Outer Membrane Modulate Antimicrobial Permeability in Klebsiella pneumoniae
Source: mBio. 2020 Apr 14;11(2):e00603-20. doi: 10.1128/mBio.00603-20 (PMC7157821; doi:10.1128/mBio.00603-20)
Supplement: TABLE S2 [file mBio.00603-20-st002.docx]

**Supplementary Table S2 – Mass spectrometry data on most abundant OMPs**

|  | **Matching uniprotID in reference strain (MGH78578)** | **Predicted molecular weight (kDa)** | **Mascot score** |
| --- | --- | --- | --- |
| Trx | A6T5E4 | 33.5 | 11829 |
| OmpA | A6T751 | 38.1 | 59351 |
| OmpK36 | A6TBT2 | 40.1 | 66104 |
| OmpK35 | A6T721 | 33.8 (premature stop codon in MGH78578) | 30363 |
| LamB | A6TGU6 | 47.9 | 36740 |
| TolC | A6TE24 | 53.5 | 27445 |
