# Supplementary material for: Global Trends in Proteome Remodeling of the Outer Membrane Modulate Antimicrobial Permeability in Klebsiella pneumoniae
Source: mBio. 2020 Apr 14;11(2):e00603-20. doi: 10.1128/mBio.00603-20 (PMC7157821; doi:10.1128/mBio.00603-20)

Supplementary Figure S2 - Growth analysis of porin-expressing strains.
AJ218Δ*ompK35*Δ*ompK36* expressing the indicated porins from an anhydrotetracycline-inducible promoter were cultured for up to 24 hours and cell density was monitored by measuring absorption (OD_{600}) over time. Error bars depict the standard deviation of biological triplicates. Growth rates and final cell densities of all strains were comparable.

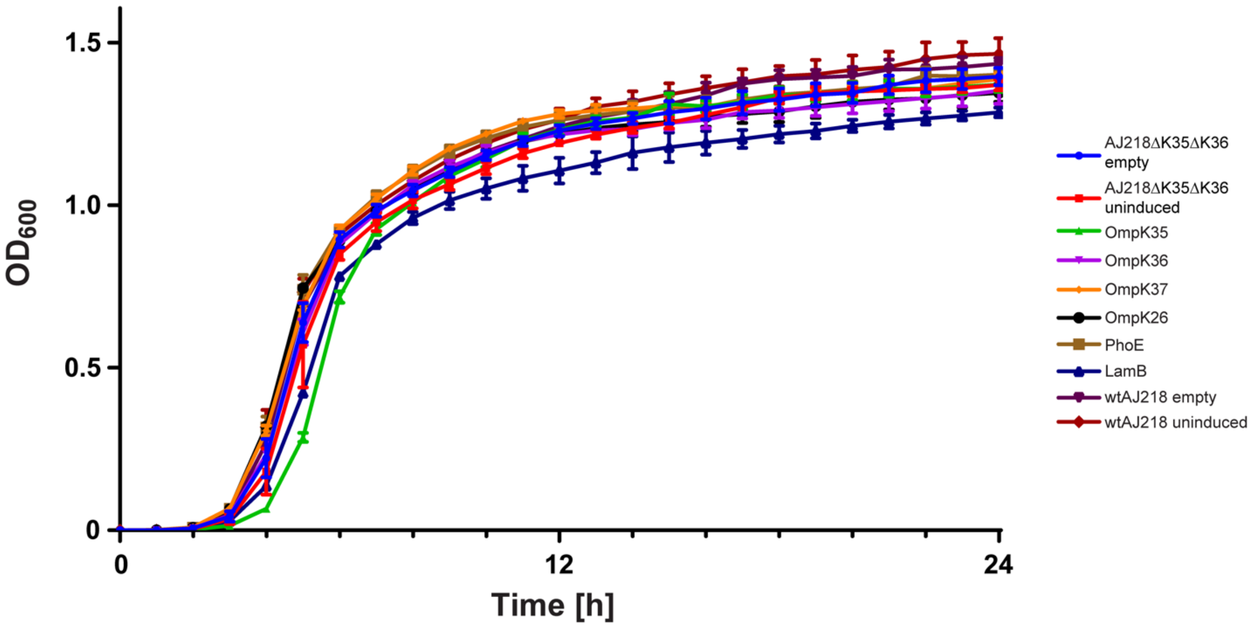

Supplement: FIG S2 [file mBio.00603-20-sf002.pdf]
