# Supplementary material for: Global Trends in Proteome Remodeling of the Outer Membrane Modulate Antimicrobial Permeability in Klebsiella pneumoniae
Source: mBio. 2020 Apr 14;11(2):e00603-20. doi: 10.1128/mBio.00603-20 (PMC7157821; doi:10.1128/mBio.00603-20)
Supplement: FIG S3 [file mBio.00603-20-sf003.docx]

**Supplementary Figure S3 – Western Blot of OmpK35 expression**. Western Blot detecting OmpK35 levels in AJ218 or AJ218Δ*ompK35*Δ*ompK36* (ΔΔ) carrying pJP-Cm, pJP(OmpK35) or pJP(OmpK36). The antibody was raised against *E. coli* OmpF, but shows cross-reactivity to *Klebsiella* OmpK35 and to a lesser degree OmpK36. Densitometry showed a 7.7-fold increase in OmpK35 expression levels in AJ218Δ*ompK35*Δ*ompK36* carrying pJP(OmpK35) compared to the wild-type strain.

**
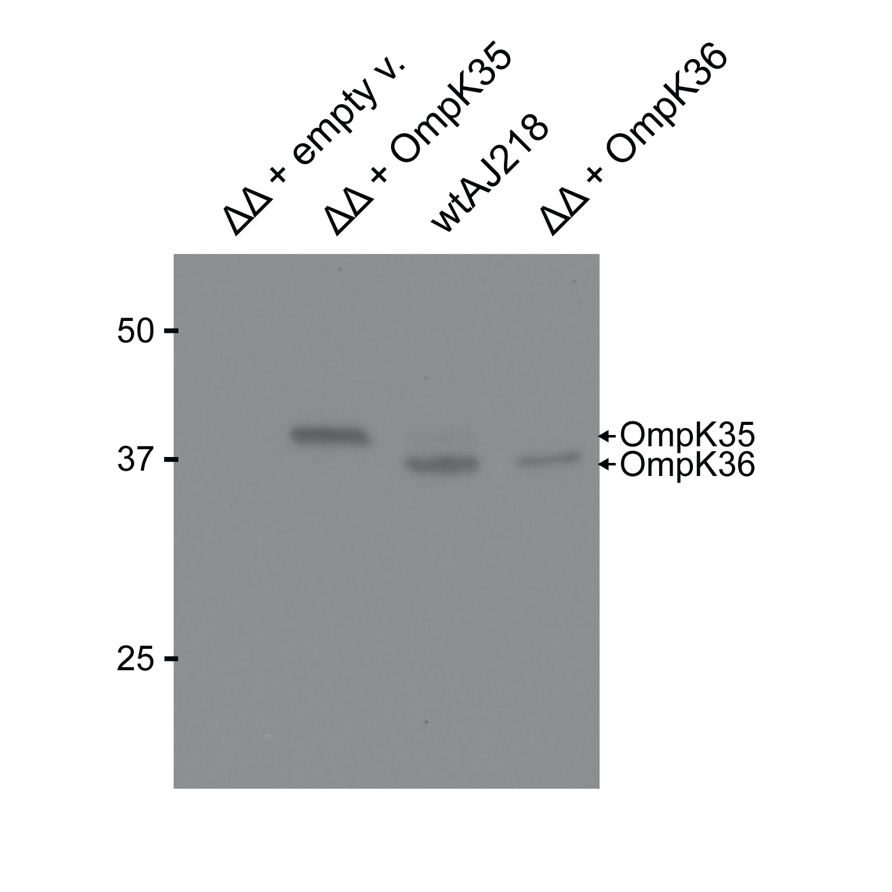
**
