# Supplementary material for: Global Trends in Proteome Remodeling of the Outer Membrane Modulate Antimicrobial Permeability in Klebsiella pneumoniae
Source: mBio. 2020 Apr 14;11(2):e00603-20. doi: 10.1128/mBio.00603-20 (PMC7157821; doi:10.1128/mBio.00603-20)
Supplement: TABLE S3 [file mBio.00603-20-st003.docx]

**Supplementary Table S3 – MIC assessments for other drug classes**

|  | **AJ218** | **AJ218Δ*K35*Δ*K36* +** | | | | | | |
| --- | --- | --- | --- | --- | --- | --- | --- | --- |
|  | **empty v.** | **empty v.** | **OmpK35** | **OmpK36** | **OmpK37** | **OmpK26** | **PhoE** | **LamB** |
| **aminoglycoside** |  |  |  |  |  |  |  |  |
| gentamicin | 0.5 | 1 | 0.25 | 0.5 | 0.5 | 0.5 | 0.5 | 1 |
| neomycin | 1 | 2 | 0.5 | 1 | 0.5 | 1 | 1 | 2 |
| tobramycin | 0.5 | 1 | 0.5 | 0.5 | 0.5 | 0.5 | 0.5 | 1 |
| spectinomycin | 128 | 256 | 32 | 128 | 256 | 256 | 256 | 256 |
| **fluoroquinolone** |  |  |  |  |  |  |  |  |
| nalidixic acid | 2 | 2 | 0.5 | 2 | 2 | 2 | 2 | 2 |
| ciprofloxacin | 0.03125 | 0.0625 | 0.004 | 0.0625 | 0.03125 | 0.0625 | 0.03125 | 0.0625 |
| **macrolide** |  |  |  |  |  |  |  |  |
| erythromycin | 64 | 128 | 32 | 64 | 128 | 128 | 128 | 128 |
| **cyclic peptide** |  |  |  |  |  |  |  |  |
| polymyxin B | 2 | 2 | 2 | 2 | 2 | 2 | 2 | 2 |
| **controls** |  |  |  |  |  |  |  |  |
| ethanol | 2.50% | 2.50% | 2.50% | 2.50% | 2.50% | 2.50% | 2.50% | 2.50% |
| isopropanol | 5% | 5% | 5% | 5% | 5% | 5% | 5% | 5% |
