# Supplementary material for: Global Trends in Proteome Remodeling of the Outer Membrane Modulate Antimicrobial Permeability in Klebsiella pneumoniae
Source: mBio. 2020 Apr 14;11(2):e00603-20. doi: 10.1128/mBio.00603-20 (PMC7157821; doi:10.1128/mBio.00603-20)
Supplement: TEXT S1 [file mBio.00603-20-s0001.docx]

**Supplementary Methods**

**Bacterial strains and cultures**

K. pneumoniae strain AJ218 (capsule serotype K54) is a human urinary tract infection isolate (1). FK688 and FK1934 were isolated from patients with bloodstream infections and were previously noted as carbapenem-resistant strains, which did not express carbapenemases (2). E. coli DH5α was used for cloning purposes. Bacterial cultures were grown in Luria-Bertani (LB) medium at 37 ºC with agitation, unless otherwise stated. For the selection of antibiotic resistance markers antibiotics were added at the following concentrations: kanamycin 30 μg/ml, chloramphenicol 34 μg/ml.

The Δ*ompK35*, Δ*ompK36* and Δ*ompK35*Δ*ompK36* mutant strains were constructed by a “gene gorging” technique as previously described utilizing ‘donor’ and helper plasmids (4, 8). All strains, plasmids and oligonucleotides used in this study are listed in Supplementary Table S3.

To construct the donor plasmids a DNA fragment was assembled by overlapping PCR containing

a kanamycin resistance cassette with flanking fragment length polymorphism (FLP) recombinase target (FRT) sites amplified from pKD4 (3). This cassette was flanked by two 500 bp genomic regions upstream and downstream of the porin genes, respectively, and designed to contain I-*Sce*I- endonuclease recognition sides on either end. The resulting DNA fragments were cloned into pGEM-T Easy (Promega) and confirmed by sequencing. pACBSR carrying L-arabinose inducible I-*Sce*I endonuclease and the genes required for lambda Red recombination was used as a helper plasmid (4). Donor and helper plasmids were transformed into *K. pneumoniae* strains by electroporation. Co-transformants were inoculated into 1 ml LB containing chloramphenicol and 0.2 % (*w/v*) L-arabinose and grown over night at 37 ºC under constant shaking. Knock-out strains were isolated as single colonies on LB-agar containing kanamycin and confirmed by PCR screening. Strains were grown in LB with 0.2% (w/v) L-arabinose without antibiotic selection to induce the loss of pACBSR. The kanamycin cassette was excised by FLP-FRT recombination using pFLPBSR, in which the I-*Sce*I endonuclease of pACBSR is replaced with the FLP recombinase from pCP20 (5, 9).

**Plasmid construction**

The coding sequence of OmpK37 was amplified from the genomic DNA of AJ218 using Phusion High-Fidelity DNA polymerase (NEB) and the primer pair K37_NcoI_f/ K37_XhoI_r, restriction digested and ligated into a pET20b vector modified to contain an N-terminal, TEV-cleavable His_10_-tag to yield the vector pET20b(OmpK37) (7). For experiments based on expression in *K. pneumoniae* strains, the ampicillin resistance marker of the anhydrotetracycline (ATc)-inducible expression vector pJP168 (6) was replaced with the chloramphenicol cassette of pKD3 (3) giving pJP-CmR. The coding sequences of the different porin genes were amplified from AJ218 genomic DNA using Vent DNA polymerase (NEB). The resulting DNA fragments and pJP-CmR were subjected to restriction digest using *Nco*I-HF and *Hind*III-HF (NEB) and ligated. The porin expression plasmids were confirmed by sequencing and transformed into *K. pneumoniae* via electroporation. Where indicated, and in all optimization experiments, the parental plasmid was use as a control.

**OmpK37 expression and purification**

For protein overexpression and purification, pET20b(OmpK37) was transformed into *E. coli* C41 cells. Bacterial cultures were grown in 0.4% glucose-containing terrific broth supplemented with ampicillin to an OD_600_ of 1 at 37 °C. Protein expression was induced by addition of 0.2 mM isopropyl-β-D-thiogalactopyranoside and cultures were grown overnight at 20 ᵒC. Cells were harvested by centrifugation (6,000 x g, 4 ᵒC, 15 min). Cell pellets were resuspended in Lysis Buffer (50 mM Tris, 200 mM NaCl, 2 mM MgSO_4_, pH 7.9) with added 0.05 mg/ml DNase I, 0.1 mg/ml lysozyme and cOmplete, EDTA-free protease inhibitor cocktail (Roche) and the mixture was incubated at room temperature for 1 hour. Cell walls were broken using a cell disruptor (Emulsiflex C3, Avestin) and the lysate was cleared by centrifugation before collecting the membrane fraction by high-speed centrifugation (100,000 x g, 4 ᵒC, 1 h). The pelleted membranes were homogenized in 45 mL Nickel Binding Buffer (50 mM Tris, 500 mM NaCl, 20 mM imidazole, pH 7.9) with 5 mL 50 % ELUGENT Detergent Solution (Sigma) and subsequently clarified by centrifugation (27,000 x g, 4 ᵒC, 10 min). The membrane fraction was loaded on a HisTrap column (GE Lifesciences) equilibrated with Nickel Binding Buffer containing 0.03 % *n*-dodecyl β-ꓓ-maltoside, washed with Washing Buffer (50 mM Tris, 500 mM NaCl, 150 mM imidazole, 0.03 % *n*-dodecyl β-ꓓ-glycoside, pH 7.9) and eluted with Nickel Gradient Buffer (50 mM Tris, 500 mM NaCl, 1 M imidazole, 0.03 % *n*-dodecyl β-ꓓ-maltoside, pH 7.9). Protein containing fractions were concentrated and loaded onto a 26/600 Superdex 200 (GE Healthcare) size-exclusion column equilibrated with SEC Buffer (50 mM Tris, 200 mM NaCl, 0.03 % *n*-dodecyl β-ꓓ-maltoside, pH 7.9). To exchange detergent, the eluted protein was bound to a HisTrap column equilibrated with OG Binding Buffer (50 mM Tris, 200 mM NaCl, 0.8 % *n*-octyl β-ꓓ-glucoside, pH 7.9), washed, eluted with OG Elution Buffer (50 mM Tris, 200 mM NaCl, 1 M imidazole, 0.8 % *n*-octyl β-ꓓ-glucoside, pH 7.9) and dialysed against OG Binding Buffer containing 1 mM dithiothreitol. The polyhistidine tag was cleaved off by overnight incubation with 0.5 mg/ml TEV protease. The solution was then passed through a HisTrap column equilibrated with OG Binding Buffer, with the flow-through containing purified TEV-cleaved OmpK37, which was concentrated to 7 mg/ml, flash-frozen in liquid nitrogen and stored at −80 °C. Protein homogeneity was monitored during all steps by Coomassie stained SDS-PAGE.

**Growth curves**

Cultures were prepared as for the outer membrane protein analysis. 200 μl of each sample were grown in triplicates in a 96-well plate using the Tecan Spark 10M. The plate was kept inside a hydration chamber at 37 ºC with orbital shaking and measurements of OD_600_ were taken every 60 minutes for 24 hours.

**Western Blot**

For detection of OmpK35 by immunoblotting, polyclonal antibodies raised in rabbits against the *E. coli* homolog OmpF were used at a dilution of 1:30,000. Densitometry was performed with the program ImageQuant TL (GE Healthcare).

**Supplementary References**

1. **Jenney AW, Clements A, Farn JL, Wijburg OL, McGlinchey A, Spelman DW, Pitt TL, Kaufmann ME, Liolios L, Moloney MB, Wesselingh SL, Strugnell RA.** 2006. Seroepidemiology of *Klebsiella pneumoniae* in an Australian Tertiary Hospital and its implications for vaccine development. J Clin Microbiol **44:**102-107.

2. **Bi W, Liu H, Dunstan RA, Li B, Torres VVL, Cao J, Chen L, Wilksch JJ, Strugnell RA, Lithgow T, Zhou T.** 2017. Extensively Drug-Resistant *Klebsiella pneumoniae* Causing Nosocomial Bloodstream Infections in China: Molecular Investigation of Antibiotic Resistance Determinants, Informing Therapy, and Clinical Outcomes. Front Microbiol **8:**1230.

3. **Datsenko KA, Wanner BL.** 2000. One-step inactivation of chromosomal genes in *Escherichia coli* K-12 using PCR products. Proc Natl Acad Sci U S A **97:**6640-6645.

4. **Herring CD, Glasner JD, Blattner FR.** 2003. Gene replacement without selection: regulated suppression of amber mutations in *Escherichia coli*. Gene **311:**153-163.

5. **Stellato MR.** 2017. Assembly and secretion of outer membrane proteins that depend on the Translocation and Assembly Module in *Klebsiella pneumoniae*. Monash University.

6. **Baldi DL, Higginson EE, Hocking DM, Praszkier J, Cavaliere R, James CE, Bennett-Wood V, Azzopardi KI, Turnbull L, Lithgow T, Robins-Browne RM, Whitchurch CB, Tauschek M.** 2012. The type II secretion system and its ubiquitous lipoprotein substrate, SslE, are required for biofilm formation and virulence of enteropathogenic *Escherichia coli*. Infect Immun **80:**2042-2052.

7. **Noinaj N, Kuszak AJ, Gumbart JC, Lukacik P, Chang H, Easley NC, Lithgow T, Buchanan SK.** 2013. Structural insight into the biogenesis of beta-barrel membrane proteins. Nature **501:**385-390.

8. **Wilksch JJ, Yang J, Clements A, Gabbe JL, Short KR, Cao H, Cavaliere R, James CE, Whitchurch CB, Schembri MA, Chuah ML, Liang ZX, Wijburg OL, Jenney AW, Lithgow T, Strugnell RA.** 2011. MrkH, a novel c-di-GMP-dependent transcriptional activator, controls *Klebsiella pneumoniae* biofilm formation by regulating type 3 fimbriae expression. PLoS Pathog **7:**e1002204.

9. **Cherepanov PP, Wackernagel W.** 1995. Gene disruption in *Escherichia coli*: TcR and KmR cassettes with the option of Flp-catalyzed excision of the antibiotic-resistance determinant. Gene **158:**9-14.
