# Supplementary material for: Global Trends in Proteome Remodeling of the Outer Membrane Modulate Antimicrobial Permeability in Klebsiella pneumoniae
Source: mBio. 2020 Apr 14;11(2):e00603-20. doi: 10.1128/mBio.00603-20 (PMC7157821; doi:10.1128/mBio.00603-20)
Supplement: TABLE S4 [file mBio.00603-20-st004.docx]

**Supplementary Table S4 – Strains, plasmids, oligonucleotides**

| ***Klebsiella* strain** | **Properties** | **Reference** |
| --- | --- | --- |
| AJ218 | Clinical isolate | (1) |
| FK688 | Clinical isolate | (2) |
| FK1934 | Clinical isolate | (2) |
| AJ218ΔOmpK35 | AJ218 with deletion of *ompK35* | This study |
| AJ218ΔOmpK36 | AJ218 with deletion of *ompK36* | This study |
| AJ218ΔOmpK35 ΔOmpK36 | AJ218 with deletion of *ompK35* and *ompK36* | This study |
| **Plasmid** | **Properties** | **Reference** |
| pKD4 | Carries a kanamycin resistance cassette with flanking fragment length polymorphism (FLP) recombinase target (FRT) sites | (3) |
| pGEM-T Easy | Amp^R^, for insertion of DNA fragments with single deoxyadenosine overhangs | Promega |
| pDonor(OmpK35) | pGEM-T Easy with kanamycin cassette flanked by *ompK35* genomic regions | This study |
| pDonor(OmpK36) | pGEM-T Easy with kanamycin cassette flanked by *ompK36* genomic regions | This study |
| pACBSR | Cm^R^, carries L-arabinose inducible I-SceI endonuclease and lambda Red recombination genes | (4) |
| pFLP-BSR | Cm^R^, carries fragment length polymorphism (FLP) recombinase | (5) |
| pJP168 | Amp^R^, for anhydrotetracycline inducible protein expression | (6) |
| pJP-Cm | Cm^R^, derivative of pJP168 with a chloramphenicol resistance marker | This study |
| pJP(OmpK35) | pJP-Cm with insertion of *ompK35* from AJ218 | This study |
| pJP(OmpK36) | pJP-Cm with insertion of o*mpK36* from AJ218 | This study |
| pJP(OmpK37) | pJP-Cm with insertion of o*mpK37* from AJ218 | This study |
| pJP(OmpK26) | pJP-Cm with insertion of o*mpK26* from AJ218 | This study |
| pJP(PhoE) | pJP-Cm with insertion of p*hoE* from AJ218 | This study |
| pJP(LamB) | pJP-Cm with insertion of l*amB* from AJ218 | This study |
| pET20bmod | Amp^R^, introduces a pelB signal sequence and a N-terminal, TEV-cleavable His_10_-tag | (7) |
| pET20b(OmpK37) | pET20bmod with insertion of *ompK37* without native signal peptide from AJ218 | This study |
| **Oligonucleotide** | **Sequence 5’→ 3’** | **Description** |
| Construction of knock-out strains | | |
| pKD4_F | tgtgtaggctggagctgcttc |  |
| pKD4_R | catatgaatatcctccttag |  |
| ompK35-upF | caaagaagactactggtggtatcg | OmpK35 donor plasmid  construction and sequencing |
| ompK35-SceF | TAGGGATAACAGGGTAATaacatcaagacctggaaatcac |  |
| ompK35-KanR | GAAGCAGCTCCAGCCTACACAgccagaatattgcgcttcatc |  |
| ompK35-KanF | CTAAGGAGGATATTCATATGgctggacgacaacgattacac |  |
| ompK35-SceR | TAGGGATAACAGGGTAATtttaccgaacagcagctcctg |  |
| ompK35-downR | gtagtatgcgtattcgcgcac |  |
| ompK36-upF | ctggcagtataaaggctaatggc | OmpK36 donor plasmid  construction and sequencing |
| ompK36-SceF | TAGGGATAACAGGGTAATccactgatggcctaattgattg |  |
| ompK36-KanR | GAAGCAGCTCCAGCCTACACAtaaccctctgtttgttatatgcc |  |
| ompK36-KanF | CTAAGGAGGATATTCATATGctacaaaatcaacctgctggatg |  |
| ompK36-SceR | TAGGGATAACAGGGTAATagcgacagggtgacgatatc |  |
| ompK36-downR | tgccgctctgattaataacctg |  |
| Cloning of OmpK37 for overexpression | | |
| K37_*Nco*I_f | gactccatggcggaaatttataataaag |  |
| K37_*Xho*I_r | gactctcgagtcagaactggtaaacc |  |
| Construction of pJP-Cm | | |
| pJP_f | gttattgtctcatgagcggatac | PCR amplification of fragments for Gibson assembly |
| pJP_r | ctgtcagaccaagtttactc |  |
| CmR_f | TCCGCTCATGAGACAATAACcttcggaataggaacttc |  |
| CmR_r | GAGTAACTTGGTCTGACAGgttgatcggcacgtaag |  |
| Cm_minusNco_f | ccccgttttcacaatgggcaaatattatac |  |
| Cm_minusNco_r | gtataatatttgcccattgtgaaaacgggg |  |
| Cloning of porins for anhydrotetracycline-inducible expression | | |
| OmpK37_*Nco*I_f | gcttccatggggaaaagaaaagtaccggc |  |
| OmpK37_*Hind*III_r | ggataagcttcatcagaactggtaaacc |  |
| OmpK35_*Nco*I_f | gcaaccatggggaagcgcaatattctggc |  |
| OmpK35_*Hind*III_r | ggataagcttcagattagaactggtaaacg |  |
| OmpK36_*Nco*I_f | gctaccatggggaaagttaaagtactgtccc |  |
| OmpK36_*Hind*III_r | catgaagcttgcaacttagaactgg |  |
| PhoE_*Nco*I_f | gcatccatggggaaaaagagtactctggc |  |
| PhoE_*Hind*III_r | gcataagcttaatcagaactggtaggtc |  |
| LamB_*Nco*I_f | gcatccatggggattactctgcgcaaacttcc |  |
| LamB_*Hind*III_r | gcataagcttaccaccacacttccatc |  |
| OmpK26_*Nco*I_f | ggtaccatggggttaaaacgctctctggttc |  |
| OmpK26_*Hind*III_r | gactaagcttcagaacgagtaagccacc |  |
